# Association between Subjective Body Image, Body Mass Index and Psychological Symptoms in Chinese Adolescents: A Nationwide Cross-Sectional Study

**DOI:** 10.3390/healthcare9101299

**Published:** 2021-09-29

**Authors:** Yueyun Zhang, Baozhong Liu, Long Sun

**Affiliations:** 1 Department of Sociology, School of Philosophy and Social Development, Shandong University, Jinan 250100, China; zyueyun@sdu.edu.cn; 2 Institute of Sociology, Chinese Academy of Social Sciences, Beijing 100732, China; liubz@cass.org.cn; 3 Center for Health Management and Policy Research, School of Public Health, Cheeloo College of Medicine, Shandong University, Jinan 250012, China; 4 National Health Commission of China, Key Laboratory for Health Economics and Policy Research, Shandong University, Jinan 250012, China

**Keywords:** subjective body image, body mass index, psychological symptoms, adolescents, cross-sectional study, China

## Abstract

Background: Conflicting findings were reported about the associations between subjective body image (SBI), body mass index (BMI) and psychological symptoms in China and other countries in the world. In this study, we aim to explore the associations between SBI, BMI, and psychological symptoms based on a large-scale, national wide survey among Chinese adolescents. Methods: The 2014–2015 China Education Panel Survey (CEPS) database, with 8134 middle school students (4137 boys and 3997 girls), was analyzed to explore the association between SBI, BMI and psychological symptoms. SBI was assessed by one question about the perception of own body shape with options “very thin”, “slightly thin”, “average”, “weak heavy”, and “very heavy”. BMI was calculated by the self-reported body weight and height. Psychological symptoms were evaluated by 10 items involving both aspects of depression and anxiety. Results: The results indicated that both boys and girls who perceived weak or very heavy weight were positively associated with psychological symptoms (*p* < 0.05). For boys, perceiving very thin body image was also in higher risk of psychological symptoms (*p* < 0.05), after controlling social-demographic variables and BMI. Comparing with normal weight boys or girls, obese boys (β = −2.22, 95% CI −3.37~−1.07) and overweight girls (β = −1.03, 95% CI −2.01~−0.06) were in lower levels of psychological symptoms after controlling for SBI. Other factors associated with psychological symptoms were family economic status, academic performance, and self-rated health status. Conclusions: A deviation from an “average” SBI was positively associated with psychological symptoms, which should be scanned when evaluating the Chinese adolescents’ mental health. These findings provide epidemiological evidence for the association between SBI and psychological symptoms in non-western social contexts.

## 1. Background

In recent decades, both overweight and obesity have been recognized as the major global public health issues with a high prevalence and burden in the world [1]. Generally, the established health risk models have identified that overweight and obesity were major risk factors for cardiovascular diseases, diabetes, musculoskeletal disorders, hormonal disorders, and certain cancers [2,3]. Furthermore, studies also indicated the association between overweight, obesity and mental problems, such as depression [4], anxiety [5] and posttraumatic stress disorder [6].

Subjective body image (SBI), which was defined as “the figuration of our body formed in our mind” [7], was also supported to be associated with mental health problems in various studies [8,9,10]. Meanwhile, as SBI was largely based on the actual level of BMI, there was a strong association between BMI and SBI [11]. Recently, the overall association between SBI, BMI and psychological problems were discussed in different studies, but with conflicting results [12,13,14]. For example, certain studies indicated that self-perceiving as extremely lean were associated with a higher level of depression symptoms [15,16,17], whereas other studies found that a larger perceptual body size and higher BMI contributed to higher level of depression symptoms [18,19]. To confound the picture further, several studies additionally found that perceived bodyweight might be more important than the actual weight in terms of increased likelihood of suicidal behavior among adolescents [20].

In addition, despite considerable discussion worldwide, little systematic knowledge about the psychological implications of both BMI and SBI was reported in China, which has been well known not just for its large population, but also for its cultural tradition deeply rooted in Confucianism. In Confucian culture, the building of a moral personality, not the building of a physical body, is usually stressed [16]. This may lead people to care less about their body image. For similar reasons, this may also weaken the association between SBI and psychological symptoms. Besides, considering the inconsistent findings of the associations among SBI, BMI and psychological problems, direct empirical evidence from China may enrich our understanding about this question in non-western societies and further serve for cross-national comparison purposes.

In the current study, the authors aim to examine the association between BMI, SBI and psychological symptoms in Chinese adolescents, using a national-wide cross-sectional dataset from the China Education Panel Survey (CEPS). To detect gender disparities, analyses were conducted separately for boys and girls throughout the study.

## 2. Methods

### 2.1. Subjects

Data were obtained from the China Education Panel Survey (CEPS), which is a nationwide panel survey of junior high school students [21]. The CEPS has been conducted annually by the National Survey Research Center at the Renmin University of China since its baseline survey during the 2013–2014 academic year. A stratified, multistage sampling with probability proportional to size was employed. First, 28 primary sampling units (PSUs) were selected from 2870 county-level districts; next, 4 middle schools were selected in each selected PSU, comprising the second-level sampling units of a total of 112 middle schools (28 units*4 schools/unit) stratified by type and size; then, in each selected school, four classes, two in grade seven and two in grade nine, were randomly sampled to form the third-level sampling units; and finally, all the students in the selected classes would become respondents. In total, a sample of 10,279 in 7th graders and 9208 in 9th graders were collected in the baseline survey. Those 9th graders, however, were not followed in later waves.

Up until now, the first two waves of CEPS have been released. Unfortunately, one of the key measures concerned in this study, subjective body image, was only collected in the second wave, thus removing the possibility of longitudinal research design. This study was therefore based upon a cross-sectional sample of Chinese adolescents in grade 8 during the 2014–2015 academic year (*n* = 9449). Excluding relevant missing data resulted in a final sample of 8134 students. As many previous studies supported the different association between SBI and psychological symptoms for boys and girls [22,23,24], this study also further explored whether the association would differ across the two gender groups.

### 2.2. Measures

#### 2.2.1. Psychological symptoms

In CEPS, the adolescents’ psychological symptoms were evaluated by 10 items about dispirited, not concentrating, unhappiness, meaningless, no energy, sadness, nervous, worried, afraid, hunching bad things, not concentrating on class, which was widely used in previous studies [25,26,27,28,29]. A 5-point scale from 1 (never) to 5 (always) was used to grade each psychological symptom, and the total scores were ranged from 10 to 50, which the higher score indicated more severe psychological symptoms. The Cronbach’s alpha of this scale was 0.91, which indicated nice reliability or internal consistency.

#### 2.2.2. Subjective body image (SBI)

Subjective body image (SBI) was assessed by asking the following question: “What do you think of your body shape?” Responses were “very thin”, “slightly thin”, “average”, “weak weight”, and “very heavy”. SBI was thus considered as an ordinal variable with values ranging from 1 (very thin) to 5 (very heavy). This instrument has been implemented in the international Health Behavior in School-Aged Children study [30,31].

#### 2.2.3. Body mass index (BMI)

BMI was constructed by self-reported body weight and body height. While self-reported weight and height might be subject to measurement error, they have been shown to be highly correlated with objectively measured height and weight [32,33]. Since the respondents in our sample were adolescents, the World Health Organization (WHO) standard was used to standardize the score by age and sex [34], and further divided it into four categories: underweight (<−2 SD), normal weight (−2~1 SD), overweight (1~2 SD) and obesity (>2 SD).

#### 2.2.4. Other factors

Other factors analyzed in this study contained students’ demographic characteristics, family background, and academic performance. Demographic characteristics included gender (boy = 1), age, whether the respondent has siblings (yes = 1) and ethnicity (Han = 0; other ethnic minority = 1). Age was a continuous variable and obtained by subtracting the year of birth from the year of the survey. Family background variables included father’s education, mother’s education, marital status of parents, and family economic status. Both father’s education and mother’s education were measured in years of completed schooling. Marital status of parents was a dummy variable, with 0 indicating “in marriage” and 1 “divorced”. Family economic status was a categorical variable with 3 values (1 = below average; 2 = around average; 3 = above average). In addition, academic performance was assessed by documented test scores in the past mid-term examination on three main subjects including Chinese, math, and English. The three scores of each student were first averaged and then further rescaled within each class to have a mean of 0 and a standard deviation of 1. 

### 2.3. Statistical Methods

Stata for Windows (version 12.0) (StataCorp, College Station, TX, USA) was used for data analysis. First, *t* test was used to compare the difference between the two gender groups on continuous variables and Chi-square tests on categorical variables. To examine the bivariate association between SBI, BMI and psychological symptoms, the ANOVA F test was employed. Second, multivariate ordinary least square (OLS) regression analysis was performed to examine the association between SBI, BMI and psychological symptoms while controlling for other covariates [35]. For each independent variable that was categorical, one category was used as the reference group and the remaining categories were included in the model as dummy variables. Finally, to account for the within-class clustering effect among students, Huber-White adjustment was employed to obtain robust estimates for standard errors and confidence intervals. All significance tests were two-tailed and a *p* value of 0.05 or lower would be considered as statistically significant.

## 3. Results

In this study, the associations between SBI, BMI and psychological symptoms were examined in Chinese adolescent. Table 1 presents summary statistics for variables included in the analysis, for the entire sample and for the boys and girls, separately. Among 8134 cases with complete information, 4137 were boys and 3997 were girls. As the table shows, the average score in the level of psychological symptoms was 21.70 (SD = 8.09). The percentages of students who rated their body image as “very thin”, “slightly thin”, “average”, “weak weight” and “very heavy” were accordingly 4.1%, 19.0%, 39.0%, 33.2%, and 4.7%. Moreover, the mean age of our final sample was 13.89 years (SD = 0.86), and nearly 55% of the respondents had at least one sibling. For more details about the social-demographic characteristics of our sample, see the first column of Table 1.

In contrasting the observed characteristics between the boy and girl students, we found that girls had a higher level of psychological symptoms, and a larger proportion of girls than boys subjectively rated their own bodies as “weak weight” and “very heavy”. Besides, gender differences are also found among most of the other covariates including basic demographics, family background, academic performance, and BMI.

Table 2 illustrates bivariate analyses between psychological symptoms and independent categorical variables and independent continuous variables, respectively. Bivariate analyses between SBI, BMI and psychological symptoms are also shown in this table. The mean score of psychological symptoms was 20.83 (SD = 7.56) among respondents with an “average” level of body image, but the corresponding score was 23.03 (SD = 8.70) for those with a “very thin” body image and 24.51 (SD = 24.51) for those with a “very heavy” body image. Moreover, in raising the level of psychological symptoms, an underestimation of body shape seemed to be more important among boys, while an overestimation of body shape more important among girls. All the factors analyzed in this study were associated with the psychological symptoms with an exception of BMI for boys (*p* = 0.222) and girls (*p* = 0.512).

Table 3 presents regression estimates regarding the effect of SBI and BMI on psychological symptoms for the entire sample and separately for each gender. As the first column of figures shows, among the entire sample, the multivariate regression estimates revealed that holding either a below-average or an above-average level of SBI posed a threat to the mental health of Chinese adolescents. For example, compared to those who rated their own body as “average”, the level of psychological symptoms for those who rated their own body as “very thin” was significantly higher (1.15, 95% CI 0.24–2.05), and the level of psychological symptoms for those who rated their own body as “very heavy” was also significantly higher (3.39, 95% CI 2.51–4.27). The right two columns of Table 3 further showed corresponding regression estimates separately for each gender. Although holding an above-average level of SBI promoted the level of psychological symptoms for both genders, the positive effect of holding a below-average level of SBI on psychological symptoms was only found among boys, but not among girls. The results show that there is statistical significance between obesity and psychological symptoms among boys, and the significance is also found for the relation between overweight and psychological symptoms among girls.

## 4. Discussion

In the current study, the associations between BMI, SBI and psychological symptoms were analyzed in Chinese adolescents. The results revealed that holding an above-average level of SBI are positively associated with more psychological symptoms for boys and girl, and holding a very thin body image was positively associated with more psychological symptoms for boys. Obese boys and overweight girls were in lower risk of psychological symptoms after controlling for SBI.

Our results supported that boys who rated their body as deviating from the average shape were in higher levels of psychological symptoms. In previous studies, the findings about the association were inconsistent. While certain studies found that perceived lean body was positively associated with psychological symptoms [36,37,38], others supported that perceived heavy body was positively associated with psychological symptoms [19,39]. There were also studies indicating that boys who viewed themselves as either underweight or overweight reported higher levels of depression, which is consistent with our study [40]. For boys who rated their body as below average (very thin or slight thin), they may experience muscle dysmorphia, which is a risk factor for mood disorders [41]. For boys who rated their body as above average (weak weight or very heavy), they may experience elevated symptoms of eating pathology, which are common comorbidity with mood disorders [42].

This study also found that girls who rated their body as above normal were in higher level of psychological symptoms. Findings about these relations in previous studies were contradictory. For example, several studies supported that perceiving heavy body was associate with psychological symptoms [43,44,45], but other studies observed the opposite [15]. Additionally, a previous study did not observe any associations [46]. Although most publications are consistent with our results [47,48], the association of psychological symptoms with “thin” and “very thin” body imaging among girls were not supported in our studies. The reason may be explained by the worldwide diffusion of the thin ideal, which may weaken the association between perceived thin body shape and psychological symptoms [49]. The self-rated body shapes with statistical significance may be also explained by the weight stigmatization in society [50].

In the current study, BMI is another important factor that merits attention. After controlling for SBI, our results supported that obese boys and overweight girls were in lower levels of psychological symptoms. With inconsistent results, previous studies found BMI was negatively associated with depression [51,52], but there were studies that supported the positive association of BMI with posttraumatic stress disorder or mental health [53,54]. There are studies that found no association with anxiety and other psychological symptoms [55,56]. The reason why obese boys and overweight girls were found to be in lower levels of psychological symptoms may be related to biological factors, such as genetic association [57] or insulin resistance [58].

Other variables were also analyzed in the current study. The results support that age is not related to psychological symptoms. This may be caused by the small age range in this study. Only the subjects of adolescents in grade 8 were analyzed in the current study. Having siblings is also positively associated with psychological symptoms among girls. It is consistent with the previous studies in China [59]. Family economic burden, academic performance, and self-reported health are all associated with psychological symptoms. The results are also supported by previous studies worldwide [60,61].

There are limitations that should be considered when interpreting the findings. First and most importantly, as a cross-section study, any causal relationships cannot be inferred for the results. Second, SBI was evaluated by one question. Although it has been used to evaluate SBI in many studies, the association between SBI and psychological symptoms may be weak because of the missing evaluation of physical appearance and physically attractive in body image status scale [62]. Third, as there are numerous varieties of psychological symptoms, only 10 items of psychological symptoms were analyzed in this study. Although they are relatively common and important, a more accurate scale can be used to evaluate psychological symptoms in the future study. Fourth, as there are many factors associated with obesity, such as hormonal disorders, thyroid stimulating hormone (TSH), free T4, free T3, adrenocorticotrophic hormone (ACTH), these factors were not analyzed in this study. It may cause bias for the findings in this study. Finally, the sample analyzed in the current study was Chinese adolescents in grade 8, and the findings of the present study should be careful to be extended to other age groups.

## 5. Conclusions

Despite these limitations, this was among the first to use data from a large-scale, national survey to examine the association among SBI, BMI, and psychological symptoms in Chinese adolescents. The results indicated that a deviation from an “average” SBI was positively associated with psychological symptoms, which should be scanned when evaluating Chinese adolescents’ mental health. Obese boys and overweight girls were at lower risk of psychological symptoms, which may be explained by biological factors. These findings provide epidemiological evidence for the association between SBI and psychological symptoms in a modern Confucian society and are helpful for us to further understanding the association from cross-national perspectives.

## Figures and Tables

**Table 1:** Descriptive statistics for the total sample and the two gender-specific samples.

Variables	Mean ± SD/*n* (%)			t/χ^2^ (Boys vs. Girls)	*p*
	Total (*n* = 8134)	Boys (*n* = 4137)	Girls (*n* = 3997)		
Psychological symptoms	21.70 ± 8.09	21.51 ± 8.45	21.89 ± 7.71	−2.12 ^†^	0.034
Subjective body image				327.38 ^‡^	<0.001
Very thin	336 (4.1)	210 (5.1)	126 (3.2)		
Slightly thin	1542 (19.0)	1020 (24.7)	522 (13.1)		
Average	3169 (39.0)	1686 (40.8)	1483 (37.1)		
Weak weight	2701 (33.2)	1071 (25.9)	1630 (40.8)		
Very heavy	386 (4.7)	150 (3.6)	236 (5.9)		
BMI				209.09 ^‡^	<0.001
Underweight	582 (7.1)	324 (7.8)	258 (6.5)		
Normal weight	6248 (76.8)	2923 (70.7)	3325 (83.2)		
Overweight	916 (11.3)	608 (14.7)	308 (7.7)		
Obese	388 (4.8)	282 (6.8)	106 (2.7)		
Age (yr)	13.89 ± 0.86	13.96 ± 0.87	13.82 ± 0.85	7.06 ^†^	<0.001
Having siblings				43.76 ^‡^	<0.001
No	3670 (45.1)	2015 (48.7)	1655 (41.4)		
Yes	4464 (54.9)	2122 (51.3)	2342 (58.6)		
Ethnicity				1.42 ^‡^	0.233
Han	7480 (92.0)	3819 (92.3)	3661 (91.6)		
Other minorities	654 (8.0)	318 (7.7)	336 (8.4)		
Father’s education (yr)	10.59 ± 3.21	10.55 ± 3.25	10.63 ± 3.18	−1.17 ^†^	0.241
Mother’s education (yr)	9.98 ± 3.55	9.89 ± 3.61	10.07 ± 3.47	−2.29 ^†^	0.022
Marital status of parents				12.77 ^‡^	<0.001
In marriage	7612 (93.6)	3911 (94.5)	3701 (92.6)		
Divorced	522 (6.4)	226 (5.5)	296 (7.4)		
Family economic status				19.30 ^‡^	>0.001
Below average	1187 (14.6)	650 (15.7)	537 (13.4)		
Around average	5997 (73.7)	2963 (71.6)	3034 (75.9)		
Above average	950 (11.7)	524 (12.7)	426 (10.7)		
Academic performance	0.05 ± 0.96	−0.17 ± 1.00	0.28 ± 0.87	−21.70 ^†^	<0.001
Self-rated health				18.02 ^‡^	<0.001
Poor	509 (6.3)	236 (5.7)	273 (6.8)		
Average	2398 (29.5)	1156 (27.9)	1246 (31.2)		
Good	5227 (64.3)	2794 (66.5)	2478 (62.0)		

Note: SD refers to standard deviation. BMI means body mass index. † denotes t values based on *t* test results indicating the difference of means between the two gender groups. ‡ denotes χ^2^-values based on Chi-square test results indicating the difference of proportions between the two gender groups.

**Table 2:** Bivariate analysis between BMI, SBI, independent variables and psychological symptoms.

Variables	Total (*n* = 8134)		Boys (*n* = 4137)		Girls (*n* = 3997)	
	Mean ± SD	*p*	Mean ± SD	*p*	Mean ± SD	*p*
**Categorical variables**						
Subjective body image		<0.001 ^‡^		<0.001 ^‡^		<0.001 ^‡^
Very thin	23.03 ± 8.70		23.51 ± 9.09		22.23 ± 8.19	
Slightly thin	21.70 ± 8.15		21.76 ± 8.38		21.60 ± 7.70	
Average	20.83 ± 7.56		20.86 ± 8.03		20.80 ± 6.99	
Weak weight	22.14 ± 8.22		21.65 ± 8.76		22.47 ± 7.84	
Very heavy	24.51 ± 9.44		23.37 ± 9.40		25.23 ± 9.41	
BMI		0.567 ^‡^		0.222 ^‡^		0.512 ^‡^
Underweight	21.77 ± 8.11		21.85 ± 8.55		21.66 ± 7.57	
Normal weight	21.72 ± 7.97		21.56 ± 8.28		21.85 ± 7.69	
Overweight	21.77 ± 8.87		21.52 ± 9.16		22.27 ± 8.25	
Obese	21.13 ± 8.17		20.54 ± 8.44		22.72 ± 7.23	
Having siblings		<0.001 ^†^		<0.001 ^†^		<0.001 ^†^
No	21.02 ± 8.27		20.95 ± 8.63		21.09 ± 7.81	
Yes	22.26 ± 7.90		22.04 ± 8.23		22.46 ± 7.59	
Ethnicity		<0.001 ^†^		0.005 ^†^		<0.001 ^†^
Han	21.57 ± 8.12		21.41 ± 8.48		21.75 ± 7.21	
Other minorities	23.10 ± 7.68		22.78 ± 7.92		23.41 ± 7.44	
Marital status of parents		<0.001 ^†^		0.001 ^†^		0.001 ^†^
In marriage	21.59 ± 8.06		21.41 ± 8.42		21.78 ± 7.67	
Divorced	23.29 ± 8.37		23.31 ± 8.77		23.27 ± 8.06	
Family economic status		<0.001 ^‡^		<0.001 ^‡^		<0.001 ^‡^
Below average	23.93 ± 8.15		23.84 ± 8.49		24.04 ± 7.71	
Around average	21.45 ± 7.88		21.23 ± 8.19		21.66 ± 7.57	
Above average	20.47 ± 8.82		20.19 ± 9.26		28.81 ± 8.25	
Self-rated health		<0.001 ^‡^		<0.001 ^‡^		<0.001 ^‡^
Poor	27.23 ± 9.64		26.38 ± 10.13		27.97 ± 9.15	
Average	23.64 ± 8.00		23.57 ± 8.25		23.70 ± 7.76	
Good	20.27 ± 7.54		20.23 ± 8.04		20.31 ± 6.95	
**Continuous variables**						
Age (yr)	-	<0.001 *	-	0.003 *	-	0.008 *
Father’s education (yr)	-	<0.001 *	-	<0.001 *	-	<0.001 *
Mother’s education (yr)	-	<0.001 *	-	<0.001 *	-	<0.001 *
Academic performance	-	<0.001 *	-	<0.001 *	-	<0.001 *

Note: SD refers to standard deviation; BMI means body mass index. † denotes *p* values based on *t* test results indicating the mean differences of psychological symptoms between two categories. ‡ denotes *p* values based on one-way analysis of variance (ANOVA) test results indicating the mean differences of psychological symptoms across three or more categories. * denotes the value corresponding to Pearson correlation coefficients between psychological symptoms and each continuous factor.

**Table 3:** Multiple linear regression analyses of the association between SBI, BMI and psychological symptoms.

Variables	Total (*n* = 8134)	Boys (*n* = 4137)	Girls (*n* = 3997)
Subjective body image (Reference = Average)			
Very thin	1.15 (0.24, 2.05) *	1.49 (0.18, 2.81) *	0.58 (−0.92, 2.09)
Slightly thin	0.40 (−0.08, 0.88)	0.46 (−0.18, 1.09)	0.33 (−0.40, 1.05)
Weak weight	1.24 (0.83, 1.66) ***	0.79 (0.09, 1.50) *	1.54 (1.03, 2.04) ***
Very heavy	3.39 (2.51, 4.27) ***	2.79 (1.04, 4.54) **	3.76 (2.42, 5.10) ***
BMI (Reference = Normal weight)			
Underweight	−0.31 (−1.00, 0.38)	−0.43 (−1.44, 0.58)	0.02 (−1.00, 1.05)
Overweight	−0.59 (−1.17, −0.02) *	−0.18 (−1.05, 0.69)	−1.03 (−2.01, −0.06) *
Obese	−2.09 (−2.93, −1.25) ***	−2.22 (−3.37, −1.07) ***	−0.86 (−2.33, 0.61)
Age (yr)	0.04 (−0.17, 0.25)	0.14 (−0.17, 0.45)	−0.06 (−0.34, 0.22)
e	0.71 (0.33, 1.09) ***	0.54 (−0.02, 1.09)	0.87 (0.34, 1.41) **
Ethnic minority	0.90 (0.24, 1.56) **	0.75 (−0.19, 1.68)	1.00 (0.19, 1.82) *
Father’s education (yr)	−0.11 (−0.18, −0.04) **	−0.15 (−0.26, −0.03) **	−0.07 (−0.18, 0.03)
Mother’s education (yr)	0.06 (−0.01, 0.13)	0.09 (−0.01, 0.19)	0.03 (−0.06, 0.13)
Parents divorced	0.96 (0.27, 1.65) **	1.27 (0.10, 2.44) *	0.59 (−0.32, 1.49)
Family economic status (Reference = Below average)			
Average	−1.32 (−1.83, −0.81) ***	−1.52 (−2.27, −0.76) ***	−1.14 (−1.85, −0.42) **
Above average	−1.69 (−2.41, −0.98) ***	−1.92 (−3.01, −0.82) ***	−1.46 (−2.53, −0.39) **
Academic performance	−0.76 (−0.94, −0.59) ***	−0.51 (−0.77, −0.25) ***	−1.14 (−1.41, −0.88) ***
Self-rated health (Reference = Poor)			
Average	−3.28 (−4.02, −2.53) ***	−2.42 (−3.79, −1.04) ***	−4.04 (−5.19, −2.90) ***
Good	−6.28 (−7.00, −5.57) ***	−5.43 (−6.77, −4.08) ***	−6.99 (−8.10, −5.88) ***
Constant	26.95 (23.79, 30.11) ***	25.10 (20.46, 29.75) ***	28.63 (24.32, 32.94) ***
*R* ^2^	0.095	0.073	0.128

Note: CI means confidence interval. BMI means body mass index. *: *p* < 0.05. **: *p* < 0.01. ***: *p* < 0.001.

## Data Availability

The dataset supporting the conclusions of this article is available on the website of China Education Panel Survey (CEPS). (Hyperlink to dataset in https://ceps.ruc.edu.cn/ (accessed on 8 January 2018)).
